# Investigating socio-economic-demographic determinants of tobacco use in Rawalpindi, Pakistan

**DOI:** 10.1186/1471-2458-8-50

**Published:** 2008-02-07

**Authors:** Ali Yawar Alam, Azhar Iqbal, Khalif Bile Mohamud, Ronald E Laporte, Ashfaq Ahmed, Sania Nishtar

**Affiliations:** 1 Community Health Sciences, Shifa College of Medicine, Pitrus Bukhari Road, Sector H-8/4, Islamabad, Pakistan; 2 Heartfile, 1-Park Road, Chak Shahzad, Islamabad, Pakistan; 3 W.H.O Pakistan, National Institute of Health, Chak Shahzad, Pakistan; 4 Department of Epidemiology, University of Pittsburgh, 3512 Fifth Avenue, Pittsburgh PA, 15213, USA; 5 Ministry of Health, Block C, Federal Secretariat, Islamabad, Pakistan; 6 Heartfile, 1-Park Road, Chak Shahzad, Islamabad, Pakistan

## Abstract

**Background:**

To investigate the socio-economic and demographic determinants of tobacco use in Rawalpindi, Pakistan.

**Methods:**

Cross sectional survey of households (population based) with 2018 respondent (1038 Rural; 980 Urban) was carried out in Rawalpindi (Pakistan) and included males and females 18–65 years of age. Main outcome measure was self reported daily tobacco use.

**Results:**

Overall 16.5% of the study population (33% men and 4.7% women) used tobacco on a daily basis. Modes of tobacco use included cigarette smoking (68.5%), oral tobacco(13.5%), hukka (12%) and cigarette smoking plus oral tobacco (6%). Among those not using tobacco products, 56% were exposed to Environmental tobacco smoke.

The adjusted odds ratio of tobacco use for rural residence compared to urban residence was 1.49 (95% CI 1.1 2.0, p value 0.01) and being male as compared to female 12.6 (8.8 18.0, p value 0.001). Illiteracy was significantly associated with tobacco use. Population attributable percentage of tobacco use increases steadily as the gap between no formal Education and level of education widens.

**Conclusion:**

There was a positive association between tobacco use and rural area of residence, male gender and low education levels. Low education could be a proxy for low awareness and consumer information on tobacco products. As Public health practitioners we should inform the general public especially the illiterate about the adverse health consequences of tobacco use. Counter advertisement for tobacco use, through mass media particularly radio and television, emphasizing the harmful effects of tobacco on human health is very much needed.

## Background

Non communicable diseases accounted for an estimated 33.4 million deaths worldwide in the year 2002; of these, 72% occurred in the developing countries [1]. Non-communicable diseases and injuries are amongst the top ten causes of mortality and morbidity in Pakistan [2]; estimates indicate that they account for approximately 25% of the total deaths within the country [3]. The most prevalent mode of tobacco use in Pakistan is smoking [4]. Smoking is an established risk factor for Cardiovascular diseases [5] and lung cancer [6]. Smoking is the single biggest modifiable risk factor for chronic disease. There are 1.1 billion smokers worldwide, 800 million smokers in developing countries [7]. By 2030 tobacco is expected to be the single biggest cause of death worldwide, accounting for about 10 million deaths per year [8]. It is pertinent to point out here that due to tighter regulations and increased taxes in Western countries, tobacco industry has shifted it's focus to developing countries like Pakistan. Aggressive marketing strategies of the tobacco industry has lead to rise in tobacco use in low income countries [9,10].

Factors which predispose the population to the risk of tobacco use need to be found out and preventive strategies devised accordingly.

The objective of this study was to investigate the socio-economic and demographic determinants of tobacco use in Rawalpindi, Pakistan.

## Methods

### Study design and Sample Size

A Cross-Sectional Survey with multi-stage cluster sampling with stratification in to rural and urban areas was done in Rawalpindi in the year 2004–2005. The survey was carried out by Heartfile (NGO working for the prevention and control of non-communicable diseases in Pakistan). The response rate for the Survey was 95%. Males and Females 18–65 years of age who were permanent residents of the area and gave informed consent, were included in the study. Main outcome measure was self reported daily tobacco use. Sample size 2018 (1038 Rural; 980 Urban), was based on an estimated prevalence of tobacco use of (20% to 30%), confidence interval 95%, precision 3% and design effect of 2.5. Questionnaire was administered through face to face interview.

### Inclusion criteria

Males and females between 18–65 years of age (inclusive), who were willing to participate and were permanent residents in the study area were included.

### Exclusion criteria

Individuals in institutionalized settings e.g. in hotels, motels, hospitals, nursing homes and other institutions and temporary residents were excluded from the study.

### Sampling Frame

Rawalpindi District was selected as the surveillance site. The choice of Rawalpindi district was made on the grounds of readily available sampling frame, logistic reasons and the fact that it is one of the big districts in Punjab province of Pakistan. In addition we wanted to initiate a mass media campaign against tobacco use in this district, after the results of this baseline survey were available. The sampling frame consisted of the entire population of Rawalpindi District. Both urban and rural areas of this district were included. There were a total of 68 Primary Sampling Units; of these 33 were in the rural and 35 were in the urban areas.

### Sampling Plan

The sampling strategy involved multi-stage cluster sampling with stratification; rural and urban areas were considered as two different strata. Each urban and rural area was further divided into clusters. In urban areas, these clusters (blocks) consisted of 200–250 structures (houses as enumerated by the Federal Bureau of Statistics) and in rural areas each village/deh/mouza was considered as a cluster. At the first stage, clusters from urban and rural areas were randomly selected proportionate to the population size of the two strata from the list of clusters. At the second stage, from each cluster a sample of 30 households was selected from each stratum using systematic sampling technique. The sampling plan was provided by the Federal Bureau of Statistics (FBS). Representatives of FBS also accompanied the field teams for identification of households. An eligible adult respondent was randomly selected from each household. Names of eligible family members of the household who were present at the time of interview were written on separate piece of papers, folded, put in a basket. After shaking the basket, one paper was drawn out by the interviewer. That person was interviewed.

### Questionnaire

Components of the questionnaire were compiled with the use of previously validated questions, as indicated below in Table 1. The questionnaire was piloted and modified as necessary. It was ensured that all questions had face validity; questions were clear, non-ambiguous and fair. Specific question regarding tobacco use was, "Whether you were using tobacco products," "Whether you were using tobacco products daily," "Type of tobacco product," "currently smoking or not," and "Oral tobacco used or not." "exposed to other people's smoke," "anyone smoke in your presence." The questionnaire was translated into Urdu and back translated in to English, ensuring consistency in phrasing of questions. The survey was carried out in Urdu language.

**Table 1:** Components of the questionnaire and their sources

**Domain**	**Method**	**Source**
Age	Date of birth. If unavailable, estimation of age with reference to an index event	Modified RISKCORN methodology [11]
Education	Based on the level of education in Pakistan	As above
Socio-economic status	Education and income	As above
Tobacco use & Smoking	Frequency and quantity Duration of exposure Past status Environmental tobacco smoke	As above INTERHEART [12]

### Defining levels of education

Levels of education as mentioned in this paper are the following: Primary (5 years of formal education), Middle (8 years of formal education), Matric (10 years of formal education), FA/FSc(12 years of formal education), Graduate and above (14 years or more of formal education).

### Ethics Committee Clearance

Ethical clearance for this study was obtained from 'Ethics committee at Heartfile,' in September 2004. Informed consent was obtained from the study participants.

### Interviewers Training

A group of 25 interviewers (graduate in social sciences/above the age of 25/both sexes) were selected for conducting interviews. One member of the field team was identified as a leader. Most data collectors had prior experience with data collection at a household level. They were trained in taking informed consent, administering the questionnaire, and interview procedures.

Quality assurance procedures were used to ensure consistency of interviewing and good quality data. Verification checks were done on 5% of the sample.

### Data Collection

Data was collected through face-to-face interviews with the help of a structured questionnaire. Informed consent was taken from the respondents before each interview. Respondents did not receive any incentives to participate.

### Data Analysis

Data were analyzed using STATA version 9.0 [13]. Statistical analysis involved summarizing data values and examining frequency distributions of all variables. Bivariate analysis was done for each variable under study in relation to daily tobacco use. Unadjusted Odds ratios and 95% confidence intervals were obtained. Family income was subdivided in to 5 quintiles (subdivision 5, 25, 50, 75 centiles). Robust multivariate logistic regression analysis was done, to take account of the cluster design effect. Variables of interest such as age, gender, rural/urban residence, family income, educational level and occupation were put in the regression model and later on forward selection method was used to find the variables with significant association with daily tobacco consumption, using the log likelihood method. The final regression model (log likelihood -707.3, p = 0.000) contained age, gender, rural/urban residence, family income and educational level. Adjusted odds ratios and 95% confidence intervals were obtained to see the association of tobacco use with various demographic and socioeconomic variables. The level of statistical significance was p < 0.05.

In order to calculate attributable percentage tobacco consumption for each level of education, adjusted proportions were used (adjusted for age, gender, rural/urban residence and family income). Adjusted proportions can be obtained using *adjust *command in stata, after regression command.

## Results

The response rate for the Survey was 95%. Mean age of the study participants was 39.4 ± 11.4 years. 841(41.7%) of the respondents were male. 1038(51.4%) of the respondents belonged to urban areas and the remaining from rural areas. 33% of the respondents had no formal education.

### Overall prevalence of tobacco use

Overall 16.5% of the study population (33% men and 4.7% women) was using tobacco on a daily basis (table 2). Among the tobacco users, the various modes by which tobacco was consumed included cigarette Smoking (68.5%), oral tobacco(13.5%), hukka (12%) and cigarette smoking plus oral tobacco (6%). Tobacco use was more prevalent in the rural areas (21%) as compared to urban areas (12.2%) (Table 2). 43.4% of tobacco users were under 40 years of age. 56% of the smokers were regularly smoking for more than 20 years, while 24% were smoking for the last 15–20 years. Among those not using tobacco products, 56% were exposed to passive smoking, out of which 35% were exposed to passive smoking daily and the rest of them few times a week. Most of the passive smokers were exposed to smoke being in the company of either the spouse (17%), co-workers (10%), friends (10%) and parents (5%).

**Table 2:** Distribution of demographic and socioeconomic variables by tobacco use (n = 2018)

Variable	**Tobacco users (n = 333) # (%)**	**Tobacco non-users (n = 1685) # (%)**	**Overall total ##3**
Residence			
Urban	127 (12.2%)	910(81.8%)	1037
Rural	207 (21%)	774 (79%)	981
Gender			
Male	277 (33%)	564 (67%)	841
Female	56 (4.7%)	1121 (95.3%)	1177
Age groups (years)			
<30	68 (14.6%)	397 (85.4%)	465
30–39	89 (14.6%)	520 (85.4%)	609
40–49	82(16.7%)	409 (83.3%)	491
50–59	76 (26.8%)	207 (73.2%)	283
>60	43 (25.3%)	127 (74.7%)	170
Income (Rupees)			
<2000	15 (21%)	57 (79%)	72
2000–3499	73 (19.2%)	307 (80.8%)	380
3500–4999	61 (17.9%)	280 (82.1%)	341
5000–8999	109 (16.3%)	558 (83.7%)	667
>9000	76 (13.6%)	482 (86.4%)	558
Education			
None	110 (17.8%)	506 (82.2%)	616
Primary	63 (19%)	270 (81%)	333
Middle	61 (21.8%)	219 (78.2%)	280
Matric	55 (14.5%)	325 (85.5%)	380
FA/FSc	27 (13.8%)	169 (86.2%)	196

Graduate & above	17 (8%)	196 (92%)	213

### Demographic & socioeconomic profile of tobacco users and non users

Table 2 presents the comparison of tobacco users and non-users in relation to various demographic and socioeconomic variables.

Table 3 (column 2 &3) present unadjusted odds ratios (95%CI) and p-values for the association of tobacco use with various demographic and socio-economic variables. Tobacco use was significantly higher in rural areas compared to urban areas, male gender compared to female, age group 50–59 years, as well as 60 years plus compared to less than 30 years age group, and uneducated population compared to higher levels of education. Family income quintiles were not significantly associated with tobacco use.

**Table 3:** Association of demographic and socioeconomic variables with tobacco use (n = 2018)

Variable (Reference Group)	**O.R (95% CI) Unadjusted**	**Unadjusted (p-value)**	O.R (95% CI) Adjusted *	**Adjusted (p-value)**
Residence				
Urban	1.00		1.00	
Rural	1.92(1.5 2.4)	**<0.001**	**1.49 (1.1 2.0)**	**0.01**
Gender				
Female	1.00		1.00	
Male	9.80 (7.2 13.3)	**<0.001**	**12.6 (8.8 18.0)**	**<0.001**
Age groups (years)				
<30	1.0		1.00	
30–39	0.97 (0.6 1.4)	0.84	0.99 (0.6 1.5)	0.97
40–49	1.1 (0.7 1.6)	0.58	0.87 (0.5 1.4)	0.55
50–59	2.2 (1.5 3.2)	**<0.001**	1.3 (0.8 1.9)	0.20
>60	2.1 (1.4 3.3)	**0.001**	0.8 (0.5 1.4)	0.45
Income (Rupees)				
<2000	1.0		1.0	
2000–3499	0.9 (0.5 1.7)	0.76	0.9 (0.4 2.2)	0.99
3500–4999	0.8 (0.4 1.6)	0.59	0.9 (0.4 2.0)	0.80
5000–8999	0.7 (0.4 1.4)	0.36	0.9 (0.4 2.2)	0.94
>9000	0.6 (0.3 1.1)	0.12	1.0 (0.5 2.4)	0.87
Education				
None	1.00		1.00	
Primary	1.54 (1.1 2.2)	0.01	0.89 (0.6 1.3)	0.51
Middle	1.62 (1.1 2.3)	**0.008**	**0.57 (0.4 0.9)**	**0.02**
Matric	0.92 (0.6 1.3)	0.65	**0.38 (0.2 0.6)**	**<0.001**
FA/FSc	0.88 (0.5 1.4)	0.59	**0.50 (0.3 0.8)**	**0.01**
Graduate & above	0.33 (0.2 0.7)	**0.002**	**0.16 (0.1 0.3)**	**<0.001**

	**95% CI = 95% Confidence Interval**			

*Adjusted for age groups, gender, rural/urban residence, family income and educational level

### Multivariate logistic regression analysis (Adjusted Associations)

Table 3 (column 4,5), present adjusted associations (Odds ratios, 95% confidence Interval and p-values) of tobacco use with various demographic and socioeconomic variables such as rural/urban residence, gender, age groups, income group and educational status. The odds ratio of tobacco use for rural residence compared to urban residence was 1.49 (95% CI 1.1 2.0, p value 0.01), being male as compared to female 12.6 (8.8 18.0, p value 0.001), middle grade education compared to No education 0.57 (0.4 0.9, p value 0.02), Matric vs No education 0.38 (0.2 0.6, p value < 0.001), FA, FSc vs No education 0.5 (0.3 0.8, p value 0.01), Graduate & above vs No education 0.16 (0.1 0.3, p value < 0.001).

### Population attributable percentage tobacco use with increasing level of education

**Figure 1:**
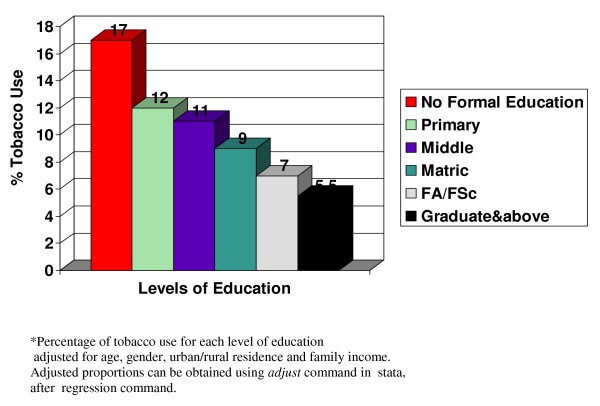
Adjusted * percentage of tobacco use across various levels of education.

Population attributable percentage of Tobacco use for each level of education was calculated (Table 4). Proportion of tobacco use at each level of education was adjusted for age, gender, urban/rural residence and family income. At first prevalence of tobacco use for the total population was calculated, using the formulas as given under Table 4. Then attributable percentage total population was calculated using the formulas shown under Table 4. No formal education was treated as the exposed group and the next level of education as the unexposed group. According to the calculation shown in Table 4, population attributable percentage of tobacco consumption increases steadily as the gap between no formal education and level of education widens. It is shown graphically in Fig 1.

**Table 4:** Attributable percentage total population for tobacco use due to lack of education

Levels of Education ↓	Population in each group %	Prevalence of tobacco use* %	Prevalence in total population† %	Attributable percentage total population $
**No Formal Education**	33	17	**-**	**-**
**Primary**	19	12	12	**0**
**Middle**	18.2	11	12	**8.3%**
**Matric**	16.5	9	12	**25%**
**FA/FSc**	8.1	7	12	**42%**
**Graduate & above**	5.2	5.5	12	**54.2%**

*Prevalence of tobacco use for each level of education adjusted for age, gender, urban/rural residence and family income. Adjusted proportions can be obtained using *adjust *command in stata, after regression command.

†Prevalence in total population = (Prevalence in exposed group × % exposed group) + (Prevalence in unexposed group × % unexposed group) (0.17 × 0.33) + (0.09 × 0.67) = 0.116 = 12%

9% is the average tobacco consumption in the educated group and 70% is the total population of educated group. Comment: No Formal education group is the exposed group

$ Attributable percentage of tobacco use in total population due to lack of education = prevalence in total population - prevalence in unexposed group

Prevalence in total population

## Discussion

### Limitations of the study

The study was done in Rawalpindi district of Pakistan, which is a big district and is comparable to other districts particularly in the Punjab province in relation to factors such as language spoken and exposure to mass media. Only 33% of the study population in Rawalpindi district had no formal education. This proportion could be higher in remote districts of Punjab and other provinces of Pakistan. The results of Rawalpindi district should therefore be read in that context. Having said that, we believe that the association of illiteracy with tobacco use, would not be different in those districts. Moreover, it is assumed, that the implications of this study in terms of public health measures would be applicable to a larger proportion of population in districts with higher prevalence of illiteracy.

We included only one person from each household in the study out of the total eligible adults in that household. This was done by simple random sampling technique and balloting in each household was done to include that person from that household. One person from each household was selected, in order to increase the total number of households surveyed and to exclude the effect of 'cluster phenomenon', where smokers tend to stick together. This could have given a false impression of high prevalence of tobacco use. However if this sampling technique has inadvertently given rise to selection bias, the results could be interpreted with caution.

### Prevalence

The overall prevalence of tobacco use in this study was 16.5%. We do not have population level data on tobacco use in Pakistan, but we do have population level data on smoking, to compare with. National Health Survey of Pakistan 1990–94, found overall prevalence of smoking as 15.2%, among those aged 15 years and above [14]. Prevalence of smoking among males in this study was 33%, other studies have also found similar results [15,16]. The predominant form of tobacco use in our study was cigarette smoking (68.5%). Oral tobacco was being used by 13.5% study participants, hukka by 12% o study participants and 6% of the tobacco users were using both cigarettes as well as oral tobacco. This highlights that the tobacco control strategies in Pakistan should not only include cigarette smoking but also oral tobacco use. Head and neck cancers are a major cancer burden in Pakistan [17]. In addition to oral tobacco use other risk factors for head and neck cancers in Pakistan are betel leaf, areca nuts, gutka and niswar [17]. Fifty eight percent of the global head and neck cancers occur in South and Southeast Asia, where chewing of betel, areca and tobacco are common [18]. The link between tobacco use and cardiovascular diseases is already established. It is important that effective steps be taken for tobacco control in Pakistan, targeting cigarettes as well as oral tobacco.

The prevalence of tobacco use among Pakistani women was quite low, 4.7% as against 33% among men, which has strong associations with social values and norms of the society. The global female smoking rates(12%) are much higher than the tobacco use among Pakistani women [19]. In Pakistan, for every 100 male smokers only 14 women smoke against 80 women in the United States [18].

Male tobacco use was 33% in our study. In another study among male adults in rural Sindh in Pakistan prevalence of current tobacco smokers was reported as high as 55% [20]. This suggests that tobacco use would vary in various districts of Pakistan depending on demographic and socio-economic profile of the population.

### Passive Smoking

Passive Smoking prevalence was very high among the study participants. Among those not smoking or using tobacco products, 56% were exposed to passive smoking, out of which 35% were exposed to passive smoking daily and the rest of them few times a week. In a study it has been reported that smoking indoors by parents had significantly higher urine cotinine levels among children as compared to smoking outdoors and also in comparison to control group of children, whose parents don't smoke [21]. Significantly greater mortality risk for never smokers living in households with smokers was reported in a cohort study. The increased mortality risk in that study has been attributed to ischaemic heart disease and cerebrovascular disease [22].

The data on passive smoking highlights the need for a concerted effort to raise awareness on this aspect of smoking and apprise smokers of their responsibilities for the protection of non-smokers health.

Awareness programs of health hazards of passive smoking should not only be limited to smokers but also to people who are affected by passive smoking such as friends, family members and co-workers. School based health education programs in this connection would be very beneficial.

### Rural Residence

Tobacco use was significantly higher in the rural areas as compared to the urban areas in this study. In another study among male adults in rural Sindh in Pakistan prevalence of current tobacco smokers was reported as high as 55% [20]. 43.7% of males aged 18 years and above were found to be smoking cigarettes in the high mountain rural areas of Pakistan [23]. This is contrary to what has been published in an earlier study in Pakistan which shows slightly higher (15.2%) prevalence of smoking in urban areas as compared to rural areas (13.7%) [18]. There is a possibility that the Tobacco Industry might be exploiting the low awareness levels about the ill effects of tobacco and smoking, in rural areas, and forcing sale of more of its products there.

### Illiteracy

Illiteracy was significantly associated with tobacco use in this study. This is consistent with other studies [14,23-27]. Tobacco use among the lower educated groups and disparity in access to health services to these groups would emerge as a major Public health threat in Pakistan. Threat to Public health would be posed as a result of increased burden of lung cancer, oral cancer and cardiovascular diseases to these disadvantaged groups. Preventing and reducing tobacco consumption among the lower educated groups should be a priority of policies aiming to reduce inequalities in health in Pakistan.

### Public Health Implications

Population attributable percentage of tobacco use increases steadily as the gap between No Formal Education and level of education widens, but this should not be inferred to be causal. We should try to entangle the factors related to education and tobacco use. Low education could be a proxy for low awareness and consumer information on tobacco products. As Public health practitioners we should inform the general public especially the illiterate about the adverse health consequences of tobacco use by community based health education programs and counter advertisement for tobacco use through mass media particularly radio and television. Periodic repetition of the informative messages would result in reinforcement of the knowledge and could translate in to long lasting behavioral change.

### Tobacco Industry

Tobacco Industry is using aggressive marketing strategies to promote smoking in developing countries [9,10,28]. Male tobacco consumption rates are declining globally while female smoking rates are increasing [19]. It has been implicated that the main targets of tobacco Industry are women and youths in these countries [19].

### Global Tobacco Research Network

Global Tobacco Research Network was developed to enhance the global tobacco control research through information sharing and collaboration among researchers [29]. It is pertinent that Pakistan should collaborate with this global Initiative to promote freedom from tobacco.

## Conclusion & Recommendations

• There was positive association between tobacco use and rural area of residence, male gender and low education levels. Low education could be a proxy to low awareness and consumer information on tobacco products.

• As Public health practitioners we should inform the adverse health consequences of tobacco use to the illiterates by community based health education programs and counter advertisement for tobacco use through mass media particularly radio and television.

• Tobacco control strategies in Pakistan should aggressively target cigarette smoking as well as oral tobacco use.

• Family members need to show extreme caution by not smoking in closed rooms in front of other family members especially children.

• We should look for ways and means to use regulations, taxes in order to make it difficult for multinationals and other retailers from selling tobacco products, especially to the illiterates.

• Restriction of smoking in public places, public transport, work places and restaurants should be ensured

## Competing interests

The author(s) declare that they have no competing interests.

## Authors' contributions

AAY: Performed data analysis and wrote the first draft of this manuscript.

IA: Involved in the designing of the survey, carrying out the survey and data acquisition.

MBK: Conceptualizing and designing the survey.

LRE: Intellectual input in writing, critical revision of the manuscript.

AA: Conceptualizing the survey and supervising the field survey.

NS: Designing, data acquisition, interpretation of results, manuscript writing.

All authors have read and agree with the contents of this manuscript. All authors have contributed in the development of this manuscript.

## Pre-publication history

The pre-publication history for this paper can be accessed here:


